# Expression profiles of lncRNAs and their possible regulatory role in monocrotaline-induced HSOS in rats

**DOI:** 10.3389/fgene.2023.1041266

**Published:** 2023-01-26

**Authors:** Mohammed Ismail, Xi Zhang, Reham Taha, Muhanad Elhafiz, Qianwen Zhang, Bashir A. Yousef, Xin Huang, Zhenzhou Jiang, Luyong Zhang, Lixin Sun

**Affiliations:** ^1^ Key Laboratory of Drug Quality Control and Pharmacovigilance, China Pharmaceutical University, Nanjing, China; ^2^ Department of Pharmacology, Faculty of Medicine and health science, Dongola University, Dongola, Sudan; ^3^ Department of Pharmacology, Faculty of Pharmacy, University of Khartoum, Khartoum, Sudan; ^4^ Centre for Drug Research and Development, Guangdong Pharmaceutical University, Guangzhou, China

**Keywords:** lncRNA, monocrotaline, HSOS, hepatotoxicity, Itpr

## Abstract

**Aims:** Long non-coding RNAs (lncRNAs) contribute to the regulation of vital physiological processes and play a role in the pathogenesis of many diseases. Monocrotaline (MCT) can cause large-scale outbreaks of toxic liver disease in humans and animals in the form of hepatic sinusoidal obstruction syndrome (HSOS). Although many experiments have been carried out to explain the pathogenesis of Monocrotaline-induced hepatic sinusoidal obstruction syndrome and to develop treatments for it, no studies have examined the role of Long non-coding RNAs in this condition. This study aimed to investigate the Long non-coding RNAs–mRNA regulation network in Monocrotaline-induced hepatic sinusoidal obstruction syndrome in rats.

**Main methods:** We established a model for MCT-induced hepatic sinusoidal obstruction syndrome, and then carried out microarray for liver tissues of SD rats in a model of early hepatic sinusoidal obstruction syndrome (12 h Monocrotaline treatment vs. control group) to investigate the differentially expressed Long non-coding RNAs and mRNAs in early hepatotoxicity. This was followed by RT-PCR analysis of selected Long non-coding RNAs, which were markedly altered. Gene Ontology (GO) and Kyoto Encyclopedia of Genes and Genome analyses were also conducted.

**Key findings:** 176 Long non-coding RNAs (63 downregulated and 113 upregulated) and 4,221 mRNAs (2,385 downregulated and 1836 upregulated) were differentially expressed in the Monocrotaline-treated group compared to the control group. The biological processes identified in GO enrichment analysis as playing a role in hepatotoxicity were positive regulation of guanosine triphosphate phosphohydrolase, liver development, and the oxidation-reduction process. Pathway analysis revealed that the metabolism pathways, gap junction, and ribosome biogenesis in eukaryotes were closely related to Monocrotaline-induced hepatotoxicity. According to these analyses, LOC102552718 might play an essential role in hepatotoxicity mechanisms by regulating the expression of inositol 1,4,5-trisphosphate receptor-1 (Itpr-1)*.*

**Significance:** This study provides a basis for further research on the molecular mechanisms underlying Monocrotaline-induced hepatotoxicity and its treatment, especially in the early stage, when successful treatment is critical before irreversible liver damage occurs.

## 1 Introduction

Long non-coding RNAs (lncRNAs) are large clusters of non-coding transcripts containing more than 200 nucleotides. lncRNAs and mRNA have an essential function in many biological processes, including the cell cycle, cell proliferation, cell differentiation, gene expression, and apoptosis (Geisler and Coller, 2013; Quinn and Chang, 2016). They act as decoys, scaffolds, and signals (Chen et al., 2019). High-throughput RNA sequencing (RNA-Seq) experiments have been conducted to identify and quantify lncRNA expression in various tissues and cell types. Generally, the expression of lncRNAs is more tissue- or cell type-specific than that of protein-coding genes, suggesting that they have distinct functions in different biological processes (Djebali et al., 2012; Holoch and Moazed, 2015; Quinn and Chang, 2016). In the domain of liver diseases, many lncRNAs have been identified, evaluated, and defined in terms of their effects on disease pathophysiology.

Monocrotaline (MCT) is a toxic retronecine-type pyrrolizidine alkaloid that occurs in a wide range of species of *leguminous Crotalaria* plants. Exposure to MCT through direct ingestion of plant matter or related foodstuffs, such as honey or milk (Roeder, 1995), has caused numerous outbreaks of poisoning in livestock and humans worldwide (Hanumegowda et al., 2003). Dehydromonocrotaline (DHM) is the toxic metabolite of MCT, which is inactivated by conjugation with glutathione (GSH) or bound to DNA-associated proteins to form pyrrole–protein adducts, causing a toxic effect (Yang et al., 2016; Boehm et al., 2017). The toxicity of MCT affects the liver, lung, and heart, causing hepatic sinusoidal obstruction syndrome (HSOS), pulmonary arterial hypertension, and right ventricular hypertrophy (Amin et al., 2011; Yu et al., 2013; Zhuang et al., 2018).

Sinusoidal endothelial cell (SEC) injury is the crucial initiating step in MCT-induced HSOS, which means that protection of SECs might prevent HSOS (Amin et al., 2011). Various studies have been conducted to explore the mechanisms underlying MCT-induced liver toxicity, including F-actin depolymerization (DeLeve et al., 1999), inflammation of the hepatic lobules, and matrix metalloproteinease 9 (MMP9), which represent the key mediators in the development of HSOS (Hanumegowda et al., 2003; Yu et al., 2013).

lncRNAs have been receiving increasing amounts of attention associated with the rapid growth of the field of molecular biology; breakthroughs in the development of new high-throughput sequencing technology, such as RNA-Seq and microarrays, have provided the basis for an improved understanding of complex transcriptomic networks and enabled us to identify the dysregulated expression of various lncRNAs in liver disease (Takahashi et al. (2014). Recently, lncRNAs have emerged as a star set of molecules that participate in a wide range of physiological and pathological processes. They have been reported to be abnormally expressed in liver diseases and have been recognized as novel biomarkers and/or key regulators of toxicological responses in human and animal models (DiStefano and Gerhard, 2022). Mild HSOS might be resolved within a few weeks, while severe syndrome is associated with multi-organ damage and a high mortality rate (>80%) (Coppell et al., 2010); for this reason, a better understanding of the pathogenesis of HSOS and the identification of earlier biomarkers could lower the incidence of complications and prevent the occurrence of potentially life-threatening severe HSOS.

Investigation of the role of lncRNAs in MCT-induced HSOS will help in the formation of a new hypothesis regarding disease pathogenesis, which will ultimately lead to new clinical applications. In this study, we performed microarray analysis on liver tissues of SD rats treated with MCT to identify the differentially expressed lncRNAs, examine their possible mechanisms in the pathogenesis of liver injury, and identify the roles of lncRNAs in HSOS; an understanding of these roles is pivotal for the ability to treat it successfully before irreversible liver damage occurs.

## 2 Materials and methods

### 2.1 Materials

Monocrotaline (CAS number 315-22-0, purity 99.48%) was purchased from Chengdu Must Bio-Technology Co., Ltd. (Sichuan, China). All other chemicals and reagents were of analytical grade.

### 2.2 Animal experiment

Sixty male SD rats, aged 7–9 weeks, were used in the experiment. These were purchased from Vital River Laboratory Animal Technology Co., Ltd. (Beijing, China). The rats were housed in standard laboratory cages under a 12 h light–12 h dark cycle and at a constant ambient temperature (20°C–25°C) and humidity (30%–50%). The rats were provided with *ad libitum* access to water and food. In order to construct a dose-dependent curve model, 30 SD rats were randomly assigned to five groups (6 rats/group). The control group was gavaged orally with normal saline, and the other groups were exposed to different doses of MCT (40, 80, 160, and 240 mg) for 24 h; after 24 h, the rats were euthanized. For the HSOS model, another 30 SD rats were randomly assigned to five groups (6 rats/group). A single dose of MCT (160 mg/kg) was administered orally to each of these groups for different periods of time (12, 24, 48, and 72 h), and the animals were subsequently euthanized, as has been previously described as a reproducible model of HSOS (DeLeve et al., 1999).

The experimental protocol was approved by the Ethics Committee of China Pharmaceutical University (Approval No. 2020-03-005) and was conducted in compliance with the guidelines of the Laboratory Animal Management Committee of Jiangsu Province.

### 2.3 Serum alanine and aspartate aminotransferase levels

Blood was collected from the rats immediately before euthanasia. Serum was obtained by centrifuging the blood at 10,000 x g for 10 min, and commercial ALT and AST kits (Whitman Biotech, China) were then used to measure the levels of alanine and aspartate transferase enzymes, following the manufacturer’s instructions.

### 2.4 Histopathological examination

Each rat’s liver was collected immediately following euthanasia. The liver was fixed in 4% paraformaldehyde and stained with hematoxylin and eosin. Histological analysis was performed using an Olympus BX-53 light microscope (Olympus, Japan).

### 2.5 Western blot

Total protein extracts were obtained by homogenizing liver tissue in RIPA buffer (Beyotime, China) supplemented with protease and phosphatase inhibitors (Thermo, United States). Cell debris was removed by centrifugation, and the supernatant was collected and stored at −80°C. Protein concentration was measured using a BCA protein assay kit (Beyotime, China), and 50 μg of protein from different samples was electrophoresed using 10% SDS–PAGE and transferred to a PVDF membrane. Immunoreactive bands were then visualized *via* enhanced chemiluminescence. The following antibodies were used in this study: cleaved caspase-9, cleaved caspase-3, cytochrome c, Bax 2772s (Cell signaling technology, United States), and β-actin as a reference protein (Santa Cruz Biotechnology, United States).

### 2.6 RT-PCR

Trizol reagent was used to extract total RNA from liver tissue in accordance with the manufacturer’s instructions. 2 µg of RNA was reverse-transcribed into cDNA using the HiScript II Q RT SuperMix for qPCR (+gDNA wiper). The SYBR Green Master Mix Kit (Vazyme Biotech, China) was used to amplify cDNA using the Step One real-time PCR system (Applied Biosystems, Thermo Fisher Scientific, United States); final results were all subsequently normalized in terms of fold changes with respect to the target gene or housekeeping gene. The primer sequences are listed in Supplementary Table S1.

### 2.7 Differential lncRNA and mRNA screening and hierarchical clustering

Liver tissues were collected from rats of the control and 12 h MCT treatment groups, and RNA was extracted. Total RNA was quantified using the NanoDrop ND-2000 (Thermo Fisher Scientific Inc., Boston, MA), and RNA integrity was assessed using the Agilent Bioanalyzer 2,100 (Agilent Technologies, Inc., Santa Clara, CA). Total RNA was transcribed to double-strand cDNA, then synthesized into cRNA and labeled with Cyanine-3-CTP. The labeled cRNAs were hybridized into the microarray. After washing, the arrays were scanned using the Agilent Scanner G2505C (Agilent Technologies). Array images were analyzed using Feature Extraction software (version 10.7.1.1, Agilent Technologies) to obtain raw data. Finally, GeneSpring (version 14.8, Agilent Technologies) was employed to complete basic analysis of the raw data. Differentially expressed genes were identified in terms of fold change, with corresponding *p*-values calculated *via t*-test. The threshold set for up- and downregulated genes was fold changes ≥2.0 and an associated *p*-value ≤0.05. Subsequently, hierarchical clustering was carried out to visualize the distinct gene expression patterns occurring among the samples; this analysis was implemented using the clustermap function of the Python package Seaborn. Arrays were run in triplicate. Agilent-“085628” was used in this experiment, and analysis of the samples was conducted by OE Biotechnology Co., Ltd. (Shanghai, China).

### 2.8 GO and KEGG enrichment analyses

Differentially expressed genes were analyzed *via* gene ontology (GO) (Ashburner et al., 2000; The Gene Ontology Consortium, 2019) to describe their functions. The number of differentially expressed genes included in each GO entry was counted, and the significance of enrichment was calculated in each case using a hypergeometric distribution algorithm (Mi et al., 2017)**.**

Pathway analysis of differentially expressed genes was performed using KEGG data, and the hypergeometric distribution algorithm was used to calculate the significance of differential gene enrichment in each pathway.

### 2.9 Co-expression analysis of differentially expressed lncRNAs and genes

A Pearson correlation test was conducted to quantify correlations between the differential expression of lncRNAs and genes. Correlated pairs with a correlation coefficient ≥0.8 and *p*-value ≤0.05 were identified.

### 2.10 Target gene analysis of trans- and cis-acting lncRNAs

In order to determine whether each lncRNA might regulate its target gene in a cis or trans manner, the RNA interaction software package RIsearch-2.0 (Alkan et al., 2017) and the FEELnc software package (Wucher et al., 2017) were applied to assess trans function and cis function, respectively. Trans function analysis was based on the use of differential co-expression results to predict the combined co-expression of candidate lncRNAs and genes at the nucleic acid level and the base binding free energy (Alkan et al., 2017). Cis function analysis was based on a search for all mRNAs within the range of 100 K upstream and downstream of differentially expressed lncRNAs and intersecting with differentially expressed genes with a significant correlation coefficient (Wucher et al., 2017).

### 2.11 Statistical analysis

Groups were compared *via* one-way analysis of variance (ANOVA) or *via* unpaired two-tailed *t*-test, when appropriate, using GraphPad Prism 6 (GraphPad Software, Inc., San Diego, CA, United States). Data are reported in the form mean ± SEM, and *p*-values <0.05 are considered to represent significance.

## 3 Results

### 3.1 Monocrotaline-induced HSOS model

**FIGURE 1:**
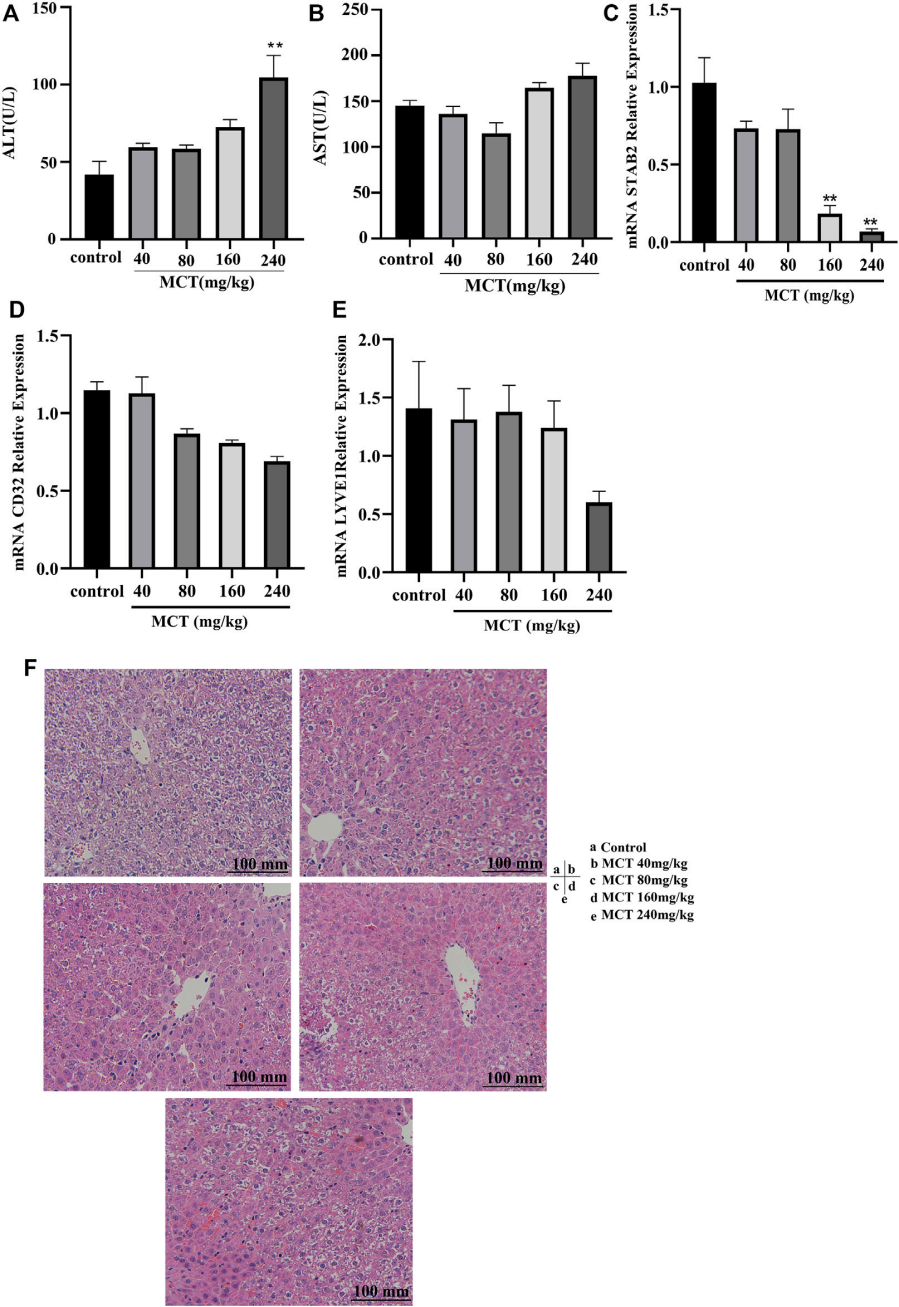
Dose-dependent monocrotaline model. Different concentrations of MCT (40, 80, 160, and 240 mg/kg) were administered orally to SD rats for 24 h. Panels show level or relative expression of **(A)** ALT, **(B)** AST, **(C)** Stab2, **(D)** CD32, and **(E)** Lyve1. **(F)** HE images for liver tissue exposed to different concentrations of MCT. MCT, monocrotaline; ALT, alanine transferase; AST, aspartate transferase enzyme; LSEC, liver sinusoidal endothelial cell; CD31, cluster of differentiation 31; Lyve1, lymphatic vessel endothelial hyaluronan receptor 1; Stab2, stabilin 2; HE, hematoxylin and eosin stain. Data are presented in the form mean ± SD (*n* = 6 rats). *p ≤ 0.05; **p ≤ 0.01; ***p ≤ 0.001 in comparisons of the MCT-treated group against the control group. Scale bars = 100 mm.

The dose-dependent model was constructed using doses of 40, 80, 160, and 240 mg/kg of monocrotaline administered orally over 24 h. The model showed an increase in liver enzymes (AST and ALT) at 160 and 240 mg/kg; however, this increase was significant only at 240 mg/kg. Histological examination results are presented in the form of HE images (Figure 1F); these demonstrate that there was no clear change in the case of 40 and 80 mg/kg doses, while extravasation of red blood cells into the perivenular space and the perisinusoidal space of Disse was clearly evident in the case of 160 and 240 mg/kg doses. Furthermore, RT-PCR of stab2 revealed a significant decrease in expression in the group receiving both 160 and 240 mg/kg (Figure 1C). In addition, CD32 and lyve-1 were decreased after MCT administration but it is not significantly (Figures 1D–E). Based on the above results, 160 mg/kg of monocrotaline was selected as the dose for establishment of the HSOS model.

**FIGURE 2:**
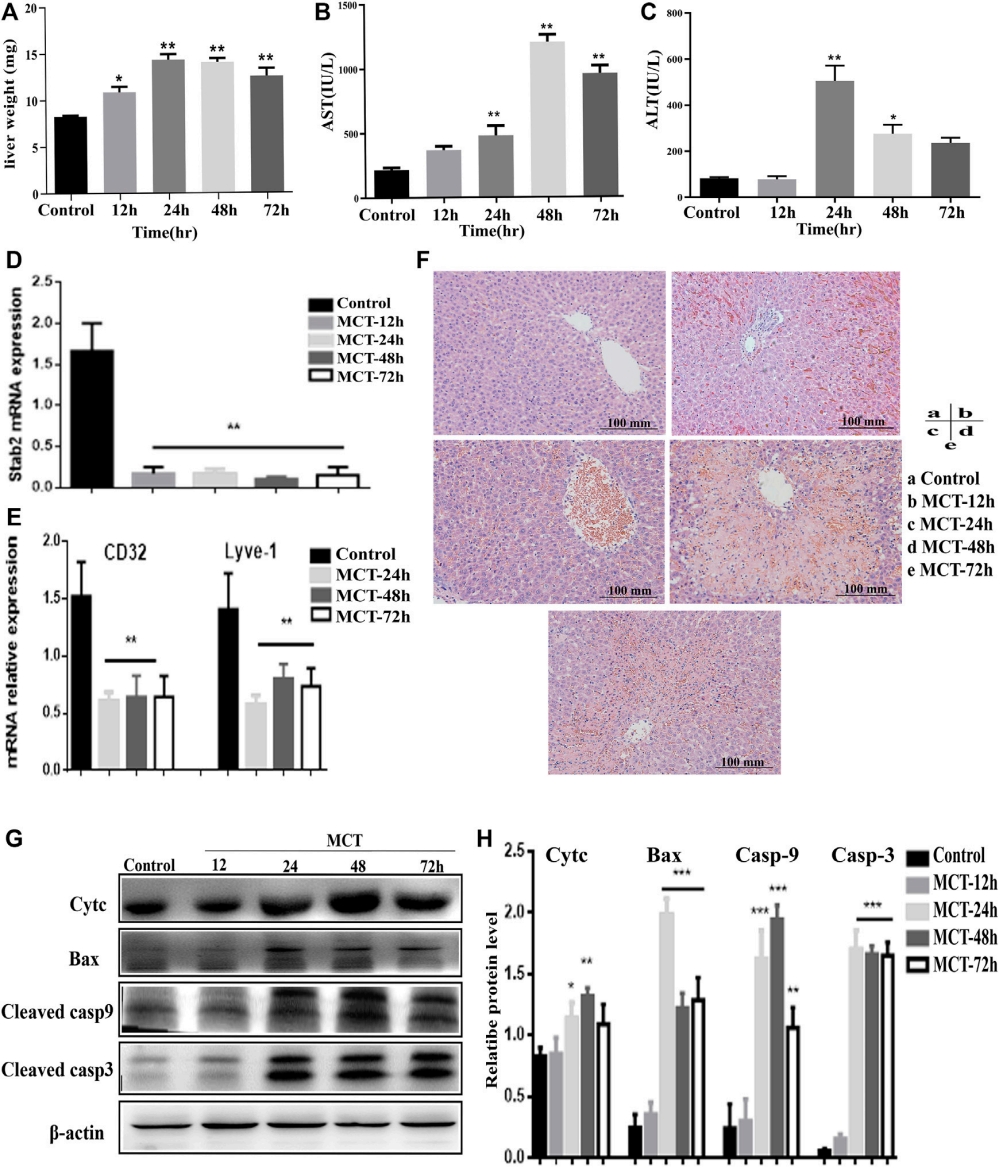
Model of hepatotoxicity induced by MCT. 160 mg/kg of MCT was administered orally to rats for different periods of time (12, 24, 48, and 72 h). Features of HSOS were observed after 48 h, while earlier features of hepatotoxicity occurred at 12 h of MCT treatment. **(A)** Change in rat liver weight after MCT treatment (normalized to total body weight). **(B,C)** Blood serum levels of liver enzymes (AST, ALT). **(D)** Relative expression of mRNAs related to LSEC-specific gene Stab2. **(E)** Relative mRNA expression of CD31 and Lyve1. **(F)** HE images of liver sections. **(G)** Western blot images indicating expression of cyt c, Bax, cleaved casp-9 and -3, and β-actin protein. **(H)** Quantification of immunoblotting results for apoptotic proteins. Data are presented in the form mean ± SD (*n* = 6 rats). *p ≤ 0.05; **p ≤ 0.01; ***p ≤ 0.001 in comparisons of the MCT-treated group against the control group. Scale bars = 100 mm.

In the second model, 160 mg/kg MCT was administered and samples were collected for analysis after different periods of time (12, 24, 48, and 72 h). Liver weight and serum ALT and AST levels were elevated at multiple time points following administration of MCT (Figure 2A). AST levels were significantly elevated after 12 h and showed time-dependent elevation (Figure 2B), while ALT level was increased significantly after 24 h of monocrotaline administration (Figure 2C). Histopathological examination of the liver tissue after 12 h revealed early signs of hepatotoxicity, such as liver inflammation and extravasation of red blood cells into the perivenular space and the perisinusoidal space of Disse, while severe hepatotoxicity signs that might indicate the development of HSOS, including loss of perivenular hepatocytes, damage to the endothelial cells of the central venule, sinusoidal hemorrhage, dilatation, and coagulative necrosis of hepatocytes, were observed after 48 h (Figure 2F). Moreover, the genetic expression of various sinusoidal endothelial cell-specific genes, such as a cluster of differentiation 32 (CD32), lymphatic vessel endothelial hyaluronan receptor 1 (Lyve1), and stabilin-2 (Stab2), was decreased in the liver tissues after MCT treatment; this further confirmed the damage to the liver SECs and induction of HSOS by MCT (Figures 2D, E). In addition to all the above effects, the levels of apoptotic proteins, such as Bax and cyt c, and cleaved caspase-3 and -9 were increased after exposure to MCT (Figures 2G, H), indicating that induction of apoptosis is a potential mechanism in this model.

### 3.2 Differential expression of lncRNAs and mRNAs during early hepatotoxicity

Microarray analysis showed that 176 lncRNAs and 4,221 mRNAs were differentially expressed among the MCT-treated group, using cut-off criteria of fold change ≥2.0 and *p*-value ≤0.05. Of the 176 lncRNAs, 113 were upregulated and 63 were downregulated, while of the 4,221 mRNAs, 1836 were upregulated and 2,385 were downregulated (Supplementary Table S2). Microarray data were deposited in the Gene Expression Omnibus database (https://www.ncbi.nlm.nih.gov/geo/query/acc.cgi?acc=GSE213031).

**FIGURE 3:**
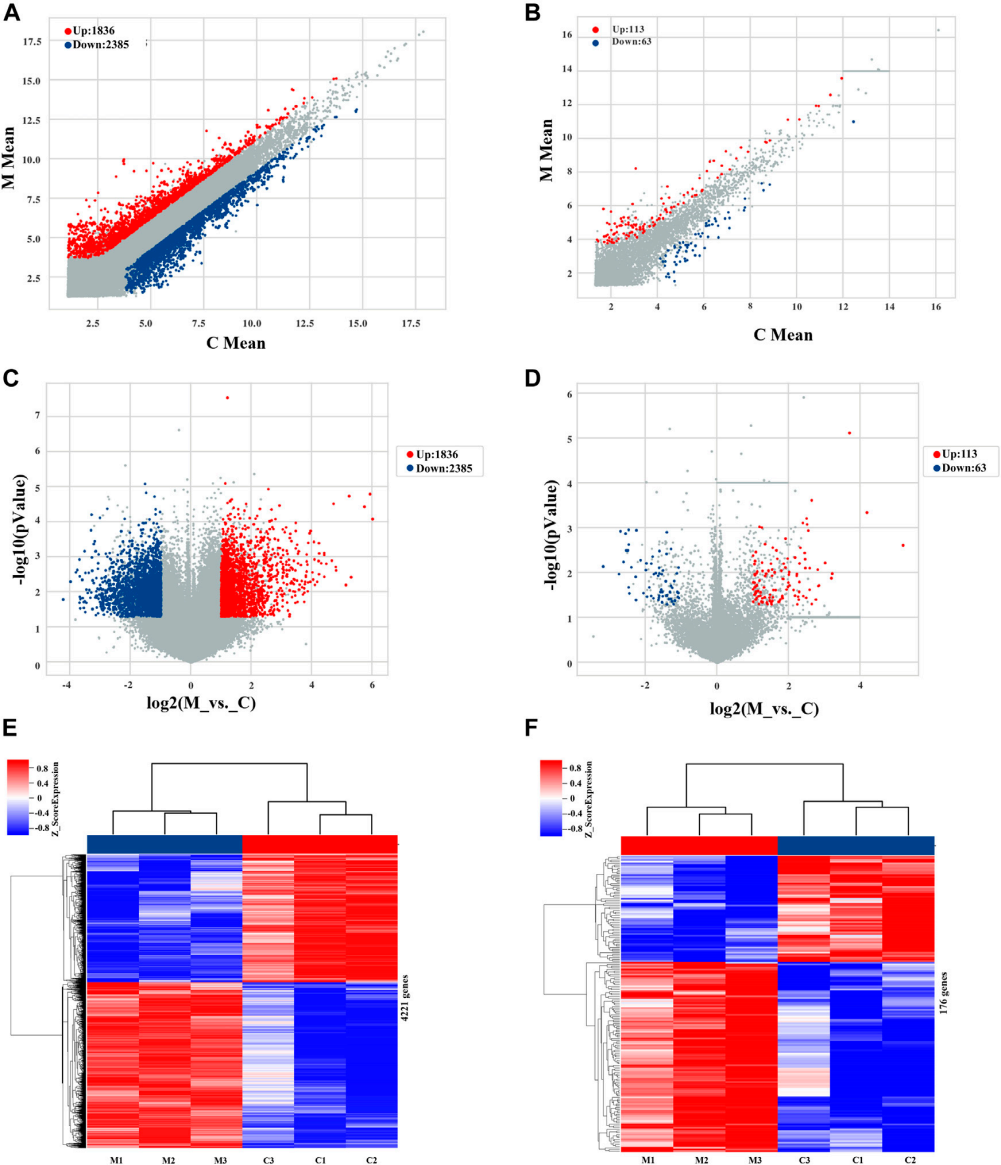
Profiles of lncRNAs and mRNAs differentially expressed during early hepatotoxicity induced by MCT. **(A,B)** Scatter plots of differentially and non-differentially expressed mRNAs and lncRNAs, respectively. **(C,D)** Volcano maps of differentially and non-differentially expressed mRNAs and lncRNAs, respectively. Each point on the graph corresponds to one mRNA/lncRNA; the abscissa corresponds to its expression in the control group, and the ordinate corresponds to its expression in the MCT treatment group. **(E,F)** Cluster heat maps of mRNAs and lncRNAs, respectively, in the control and MCT-treated groups. Each row corresponds to a differentially expressed mRNA/lncRNA, and each column corresponds to a sample. Color indicates the relative change to the corresponding mRNA/lncRNA in each sample: red corresponds to high expression, blue corresponds to low expression, and gray (in volcano and scatter plots) represents an mRNA/lncRNA that does not meet the corresponding threshold. C, control group; M, monocrotaline-treated group.

Differential expression of lncRNAs and mRNAs is presented on a scatter plot to illustrate the differences in gene expression between the two sets of data (Figures 3A, B). Additionally, a volcano plot was produced to evaluate the distribution of differences in levels of expression and corresponding *p*-values across many lncRNAs and mRNAs, as well as the distribution of differentially expressed genes after final selection of the most relevant (Figures 3C, D). Hierarchical clustering analysis illustrates the differentially expressed signatures of both lncRNAs and mRNAs (Figures 3E, F), in which distinguishable gene expression patterns are evident among the samples.

Based on the results of the gene differential expression analysis, we found that the most upregulated gene in the MCT treatment group was ATP-binding cassette, subfamily B (MDR/TAP), members 1B (Abcb1b), Abcb1b-X2, and Abcb1b-X1, with fold changes 60 (*p*-value = 0.00002), 64 (*p*-value = 0.00008), and 53 (*p*-value = 0.00004), respectively, while the most downregulated genes were glial cell line-derived neurotrophic factor receptor alpha-1 (GDNF family receptor alpha-1, transcript variant X2; Gfra1) and LARGE xylosyl- and glucuronyltransferase 1, transcript variant X1 (Large1), with fold changes of −19 (*p*-value = 0.016) and −16 (*p*-value = 0.005), respectively.

### 3.3 Validation of microarray results *via* RT-PCR

**FIGURE 4:**
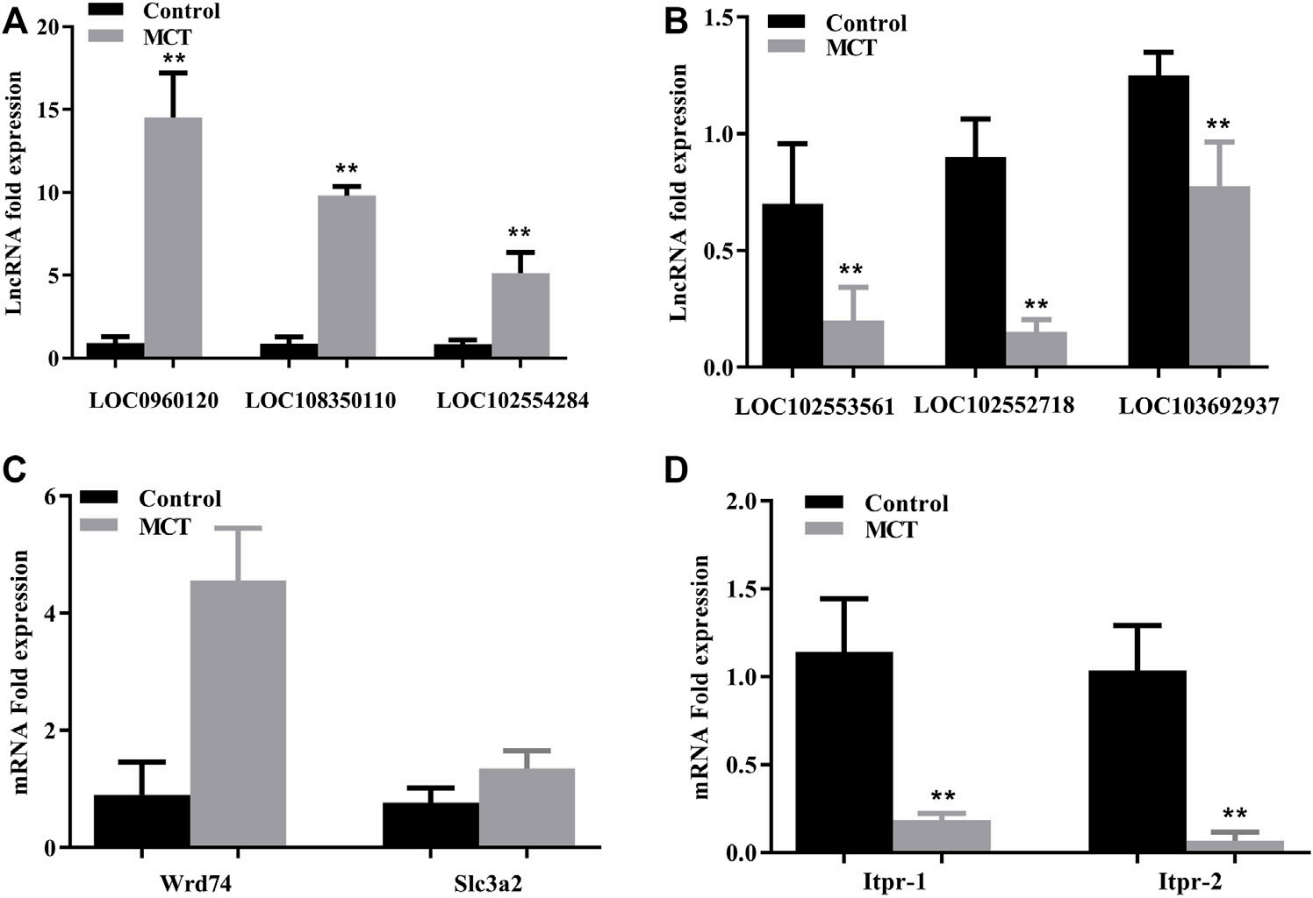
Validation of microarray results using RT-PCR. **(A)** Relative expression of upregulated lncRNAs LOC0960120, LOC108350110, and LOC102554284. **(B)** Relative expression of downregulated lncRNAs LOC192553561, LOC102552718, and LOC103692937. **(C)** Relative expression of upregulated mRNAs (Wrd74 and Slc3a2). **(D)** Relative expression of downregulated mRNAs (Itpr1 and Itpr2). *Itpr1*: inositol 1,4,5-trisphosphate receptor type 1; *Wdr74:* WD repeat domain 74; *Slc3a2:* solute carrier family three member 2. Data are presented in the form mean ± SD. *n* = 6 rats per time point. *p ≤ 0.05; **p ≤ 0.01; ***p ≤ 0.001 in comparisons of the MCT-treated group against the control group.

The expression of several randomly selected lncRNAs and mRNAs was examined *via* RT-PCR in order to validate the results of the lncRNA and mRNA microarray analysis. Similar patterns of expression as in the microarray results were revealed for three upregulated lncRNAs (LOC0960120, LOC108350110, and LOC102554284) and three downregulated lncRNAs (LOC102553561, LOC102552718, and LOC103692937) (Figures 4A, B). In addition, the same changes as identified in the microarray findings were also observed for two upregulated mRNAs, namely WD repeat domain 74 (Wrd74) and solute carrier family three member two (Slc3a2), and for two downregulated mRNAs (Itpr-1 and Itpr-2) (Figures 4C, D).

### 3.4 GO and KEGG pathway analysis

**FIGURE 5:**
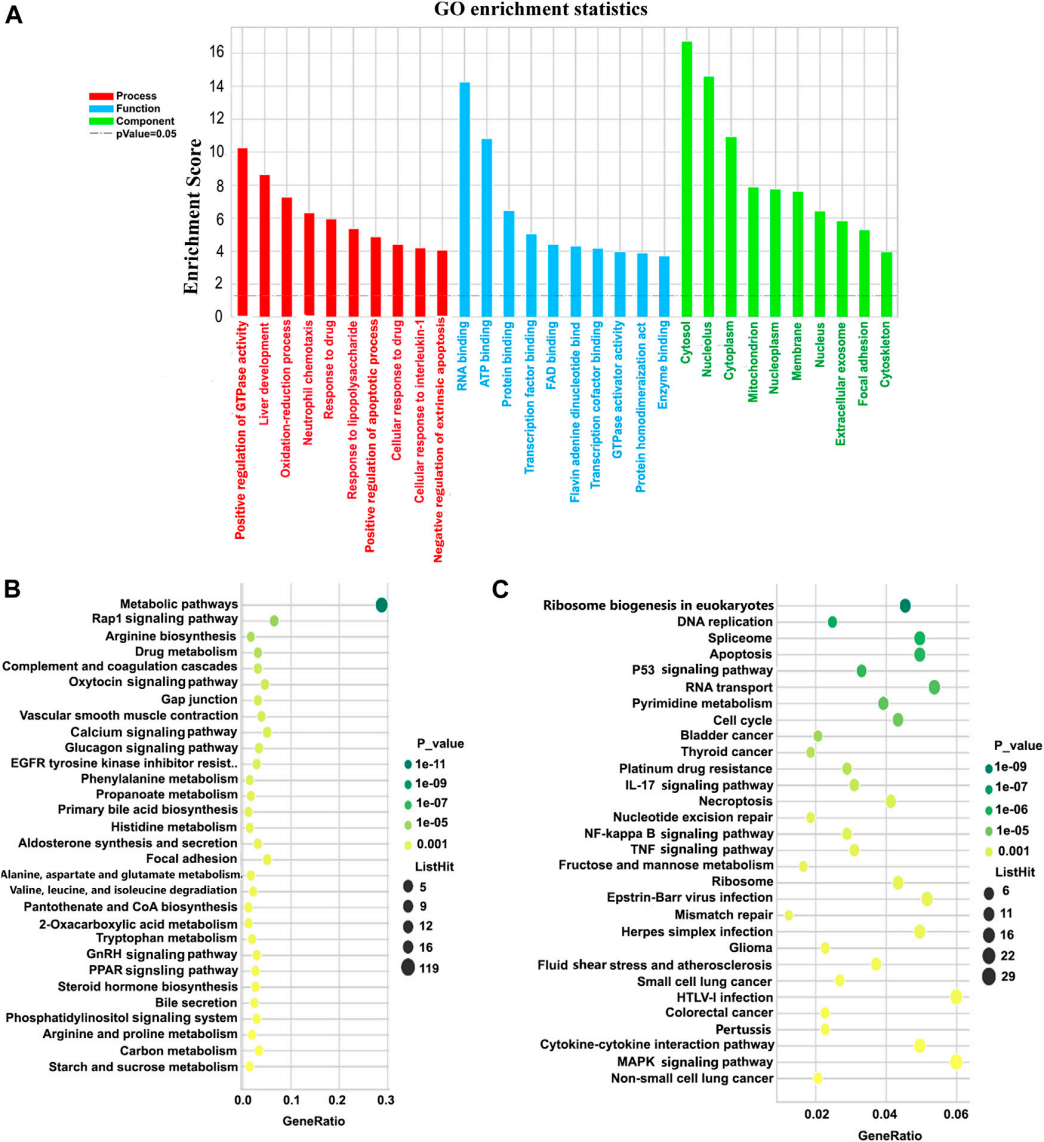
GO and KEGG pathway analyses of the biological functions of mRNAs co-expressed with differentially expressed lncRNAs. **(A)** GO enrichment for all differentially expressed mRNAs. The *Y*-axis represents -log10 (*p*-value), with higher bars therefore representing smaller corresponding *p*-values. Colors differentiate BPs, CCs, and MFs. **(B,C)** KEGG bubble charts for the top 30 pathways associated with downregulated and upregulated mRNAs, respectively. The *X*-axis represents degree of enrichment, and the *Y*-axis represents the path of enrichment. The larger the bubble, the more genes fell into this pathway, and the greener the color, the greater the significance of the enrichment. GO, Gene Ontology; KEGG, Kyoto Encyclopedia of Genes and Genomes; BP, biological process; CC, cellular component; MF, molecular function.

GO analysis of the differentially expressed genes was conducted in order to describe their functions. A total of 4,221 genes were entered into this analysis, of which 1759, 1833, and 1,694 genes were annotated for association with a biological process (BP), cellular component (CC), or molecular function (MF), respectively. Based on GO class (Figure 5A), the top 10 classifications for genes associated with a biological process among all dysregulated mRNAs were: positive regulation of GTPase activity; liver development; oxidation-reduction process; neutrophil chemotaxis; response to drugs; response to lipopolysaccharide; positive regulation of the apoptotic process; cellular response to drugs; cellular response to interleukin-1; and negative regulation of the extrinsic apoptotic signaling pathway. The top 10 molecular functions consisted of: RNA binding; ATP binding; protein binding; transcription factor binding; FAD-binding; flavin adenine dinucleotide binding; transcriptional cofactor binding; GTPase activator activity; protein homodimerization activity; and enzyme. Finally, the top 10 cellular components identified as functions of up- and downregulated lncRNAs were the cytosol, nucleus, cytoplasm, mitochondrion, nucleoplasm, membrane, nucleus, extracellular exosome focal adhesion, and the cytoskeleton.

895 genes were annotated for their KEGG pathway. Among these, there were 77 pathways with a *p*-value ≤0.05. The topmost enriched pathways corresponding to downregulated mRNAs were the metabolic process, the Rap1 signaling pathway, arginine biosynthesis, drug metabolism, complement and coagulation cascades, oxytocin signaling pathways, and gap junction (Figure 5B). The topmost enriched pathways for upregulated mRNAs were ribosome biogenesis in eukaryotes, DNA replication, spliceosome, apoptosis, the p53 signaling pathway, RNA transport, and pyrimidine metabolism (Figure 5C).

### 3.5 lncRNA–mRNA co-expression network and cis/trans pattern analysis

**FIGURE 6:**
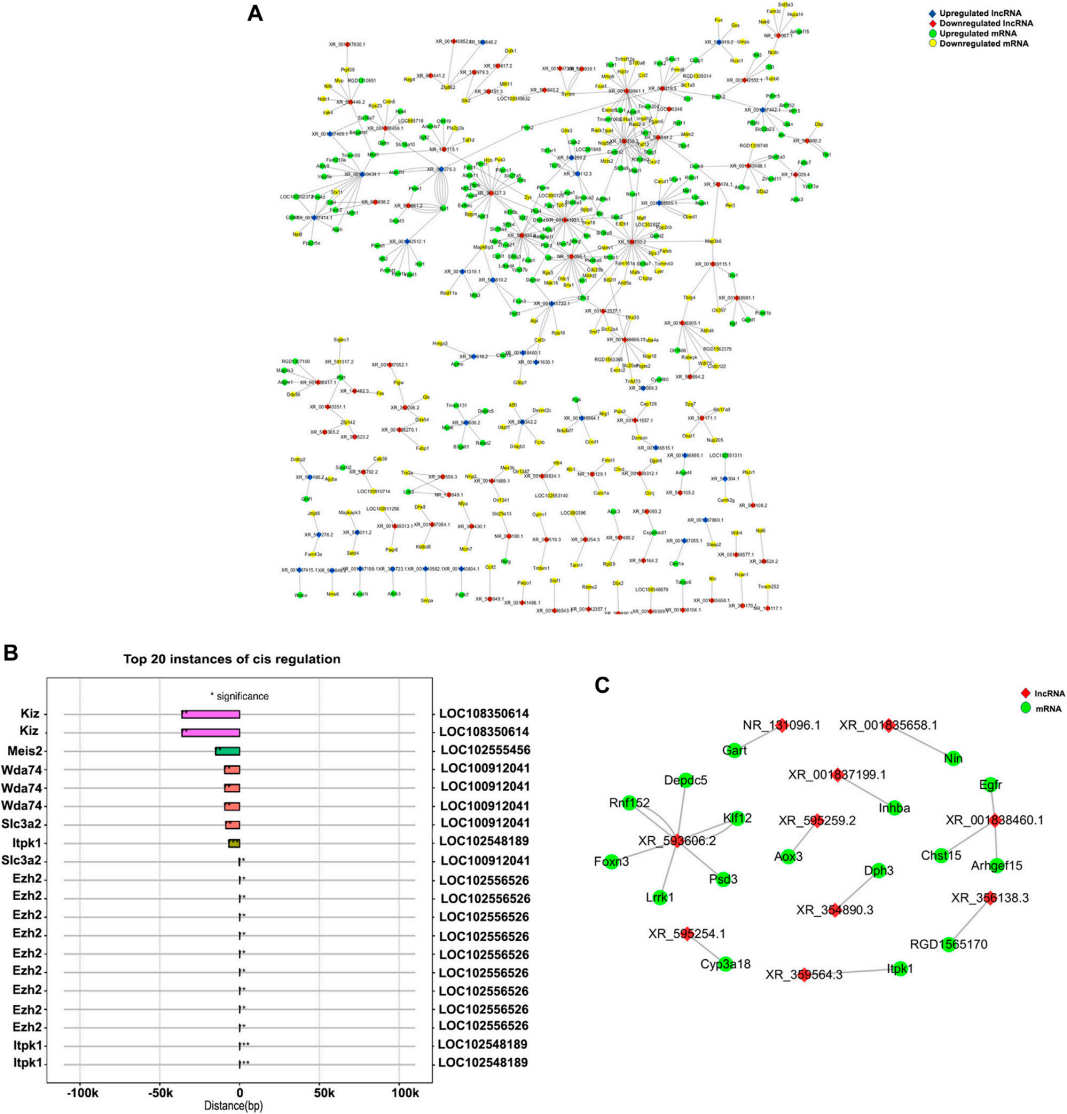
Visualization of mRNA–lncRNA co-expression network. **(A)** Network of mRNA–lncRNA co-expression drawn using Cytoscape 3.7.2. **(B)** Cis-regulated mRNA–lncRNA correlations. Number of asterisks indicates the level of significance: ***0 < *p* < 0.001; **0.001 < *p* < 0.01; *0.01 < *p* < 0.05. The left and right *Y*-axes represent mRNAs and lncRNAs, respectively; the *X*-axis represents the distance between the mRNA and lncRNA, with negative values indicating upstream distance and positive values downstream. Bars of the same color represent the same lncRNA. **(C)** Network diagram of mRNA–lncRNA trans target interaction, also drawn using Cytoscape 3.7.2. Red nodes represent lncRNAs; green nodes represent genes.

The top 500 correlations on the basis of *p*-values were identified, and Cytoscape was used to visualize the lncRNA–mRNA correlation co-expression network (Figure 6A). Interestingly, we found that LOC102552718 was co-expressed to a significant extent with Itpr-1 (correlation coefficient = 0.99, *p*-value = 0.0000007). Furthermore, co-expression data and causal interactions between these lncRNAs and their gene targets were assessed in association with information on chromosomal location (cis/trans). The top 20 lncRNA−mRNA cis interactions and trans interactions are presented in Figures 6B, C. According to the analysis, the most significant cis interaction was between LOC102548189 and Itpk1 (correlation coefficient = 0.98, *p*-value = 0.0004), with both being located on chromosome 6. In contrast, the Itpk1 gene was found to be regulated by LOC102555010 in a trans manner (correlation coefficient = 0.99, *p*-value = 0.00002, and free energy = −102.33).

### 3.6 Hypothesized regulatory role of LOC102552718 in hepatotoxicity induced by monocrotaline

**FIGURE 7:**
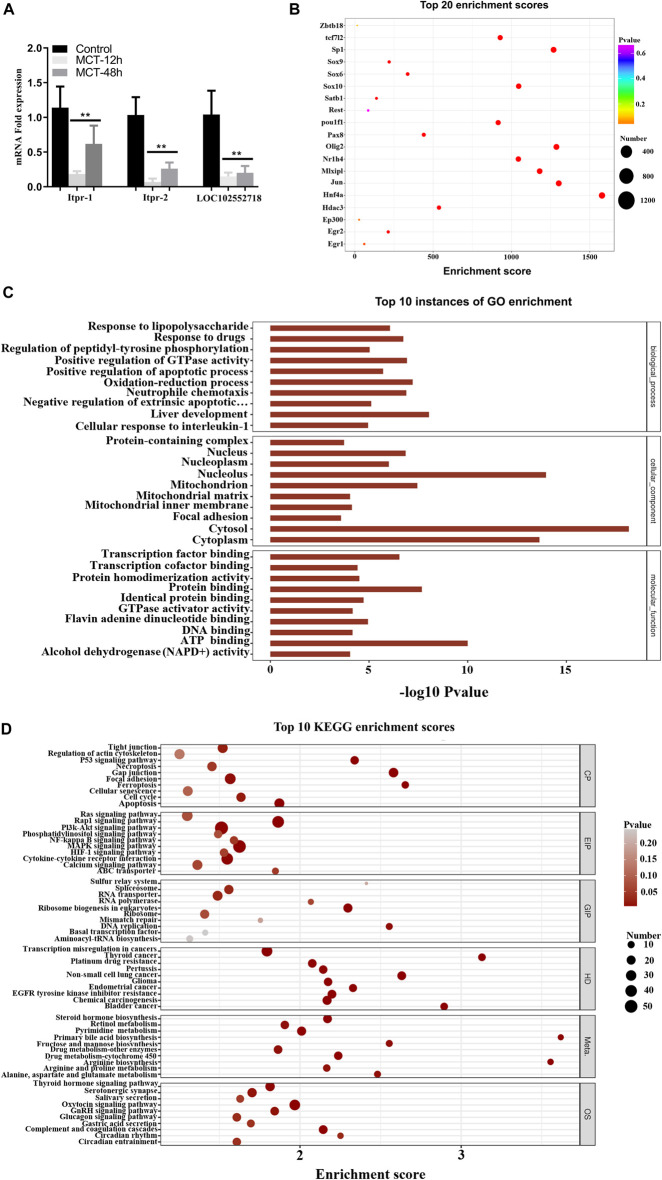
Hypothesized function of *LOC102552718* in hepatotoxicity induced by MCT. **(A)** Relative expression of Itpr1, Itpr2, and LOC102552718 in control and MCT-treated liver tissue. Data are presented in the form mean ± SD. *n* = 6 rats per time point. **(B)** Bubble chart of the top 20 enriched transcription factors associated with *LOC102552718*. The *X*-axis represents enrichment score. The larger the bubble, the higher the number of differentially expressed genes encoding the protein. Bubble color indicates the *p*-value for enrichment: the smaller the *p*-value (i.e., the greater the significance), the further the color along the scale from purple to blue to green to red. **(C)** Top 10 GO enrichment scores for *LOC102552718*. The *Y*-axis represents -log10 (*p*-value); the longer the bar, the smaller the corresponding *p*-value. **(D)** Top 10 KEGG enrichment results for differentially expressed genes co-expressed with *LOC102552718*, highlighting the top 10 entries with the smallest *p*-values for several KEGG primary classifications (Cellular Processes, Environmental Information Processing, Genetic Information Processing, Human Diseases, Metabolism, and Organismal Systems). The *X*-axis represents enrichment score. The larger the bubble, the higher the number of differentially expressed genes. Bubble color ranges from gray to red as the *p*-value representing enrichment decreases (i.e., as significance level increases). *p ≤ 0.05; **p ≤ 0.01; ***p ≤ 0.001 in comparisons of the MCT-treated group against the control group. Colors differentiate BPs, CCs, and MFs. BP, biological process; CC, cellular component; MF, molecular function.

Based on the co-expression analysis, we found that LOC102552718 might play an essential role in MCT-induced hepatotoxicity. Thus, we further explored its possible regulatory role in the model established in this study. RT-PCR results revealed that LOC102552718 was significantly downregulated following treatment with MCT (Figure 7A). Pearson correlation analysis was conducted to identify and quantify correlations between LOC102552718 and differentially expressed genes (Supplementary Table S3). Several variants of inositol 1,4,5-trisphosphate receptor type 1 (Itpr1) were found to be correlated with LOC102552718; of these, the Itpr1-X6 variant had the highest correlation coefficient of 0.99, with a *p*-value of 0.0000007. Moreover, GO and KEGG enrichment analysis was performed for each differentially expressed gene co-expressed with LOC102552718 (Figures 7B, C); these analyses revealed that gap junction and apoptosis were the most significantly enriched cellular processes, with *p*-values of 0.00001 and 0.001, respectively, while the Rap1 signaling and MAPK signaling pathways were enriched within the category of environmental information processing (EIP), with *p*-values of 0.00006 and 0.0004, respectively. Based on the gene–transcription factor pairs provided by the Gene Transcription Regulation Database (GTRD) (Yevshin et al., 2017), the differentially expressed gene corresponding to each transcription factor (TF) was calculated using the database for LOC102552718 and the clusterProfiler software package (Figure 7D). This analysis indicated that Jun proto-oncogene and the AP-1 transcription factor subunit (Jun) were highly correlated with LOC102552718.

## 4 Discussion

Liver sinusoidal endothelial cells (LSECs) have been identified as the first target of injury for some hepatotoxins, including MCT (Caldwell-Kenkel et al., 1988; McKeown et al., 1988). This can be observed clearly in the expression of Stab2, a gene related to LSECs (Figure 2D); additionally, the depletion of glutathione represents the main reason for this (DeLeve et al., 1999; Neuman et al., 2015). In the present study, we constructed a dose-dependent curve to determine the appropriate dose for the establishment of an HSOS model. This was carried out *via* oral gavage of 40, 80, 160, and 240 mg/kg of MCT in rats for 24 h. Based on the resulting HE images and liver enzymes (Figure 1), 160 mg/kg MCT was selected as the recommended dose for establishment of the HSOS model.

In this HSOS model, the levels of liver enzymes ALT and AST were found to be elevated after 12 h MCT treatment; additionally, liver inflammation and extravasation of red blood cells into the perivenular spaces appeared in the liver tissue under histopathological examination. Based on these results, hepatic sinusoidal obstruction syndrome features were observed clearly after 48 h of MCT administration. Loss of perivenular hepatocytes, damage to the endothelial cells of the central venule, sinusoidal hemorrhage, dilatation, and coagulative necrosis of the hepatocyte were observed in HE images (DeLeve et al., 2003). Furthermore, immunoblotting results for apoptotic proteins, in which cyt c, Bax, and cleaved caspase-3 and -9 levels were increased, revealed that apoptosis was induced upon treatment with MCT.

The roles played by lncRNAs in liver physiology and toxicity have been identified in various types of research. Although multiple studies have been performed to elucidate the pathogenesis of MCT-induced HSOS, no studies have been conducted on lncRNAs and their role in the pathogenesis, diagnosis, and treatment of MCT-induced hepatotoxicity. In this study, 4,221 genes and 176 lncRNAs were differentially expressed, and several biological pathways relating to these and involved in early hepatotoxicity induced by MCT have been described; additionally, the RT-PCR results showed an excellent degree of concordance with the microarray results. Furthermore, we have identified several lncRNAs that were altered during application of the HSOS model.

In order to explore these new insights into the biological and cellular mechanisms involved in MCT-induced hepatotoxicity, GO and KEGG analyses were conducted; according to the results, many pathways were activated in this model, while others were inhibited. Metabolic pathways were the most significantly downregulated (*p*-value = 1.72E-11), with many related genes being downregulated upon exposure to MCT, such as lipase, glutaminase-2, 4-hydroxy-2-oxoglutarate aldolase (Hoga1), and inositol-tetrakisphosphate 1-kinase (Itpk1). These changes in metabolic pathways may affect mitochondrial functions (the TCA cycle and ATP production), lead to an increase in the release of cyt c from the mitochondria, and activate apoptotic cascades culminating in liver cell apoptosis. Furthermore, apoptosis (*p*-value = 1.48E-06) and the TNFα signaling pathways (*p*-value = 0.00143) were upregulated in the monocrotaline treatment group.

Additionally, the calcium signaling and phosphatidylinositol signaling pathways were altered significantly in early hepatotoxicity (*p*-values = 0.000393 and 0.003046, respectively). Calcium is a crucial secondary messenger that participates in many hepatic processes. Consequently, changes in calcium signaling have been detected in mechanistically distinct liver injury conditions, and dysregulation of calcium signaling is an indicator of both acute and chronic liver diseases (Oliveira et al., 2015; Oliva-Vilarnau et al., 2018).

The extracellular matrix (ECM) provides physical protection and structural support for cells; it also regulates the adhesion, migration, differentiation, proliferation, and survival of cells (Cox and Erler, 2011). ECM production and function are altered with the development of liver disease (Friedman et al., 2000). Hanumegowda et al. have demonstrated that matrix metalloproteinases (MMPs) play a role in the endothelium damage caused by MCT (Hanumegowda et al., 2003). Generally, MMP-2, MMP-3, MMP-8, MMP-10, MMP-12, and MMP-13, among other MMPs, are significantly upregulated during hepatic IRI (Cursio et al., 2002; Hamada et al., 2008); MMP-8 appears to be among the candidates for anti-fibrotic function, and overexpression of this has been found to correlate with enhanced hepatocyte proliferation and a reduction in liver fibrosis (Iimuro et al., 2003; Harty et al., 2005; Endo et al., 2011). MMP-19 has also been found to be associated with hepatic fibrosis, and deficiency in MMP-19 has been shown to be associated with impaired TGF-β signaling and reduced liver fibrosis in the CCl_4_ murine model (Jirouskova et al., 2012).

In this study, matrix metallopeptidase-8 (MMP-8) was upregulated 3-fold in the MCT treatment group (*p*-value = 0.001624). Moreover, it was co-expressed with LOC102554284 (nucleolar protein 56-like), with a correlation coefficient of 0.999,026 (*p*-value = 0.00000146). Based on the above studies and information, LOC102554284 could represent a novel regulator for MMP-8 and a target to enhance liver cell proliferation; however, further experiments would be required to confirm this hypothesis.

The functional roles of individual lncRNAs and their downstream causal implications for responses to MCT-induced hepatotoxicity have been examined here using lncRNA–mRNA co-expression analysis, which has provided a basis for future hypothesis-driven experimental studies. LOC102552718 was one of the most downregulated lncRNAs following MCT treatment. LOC102552718 is an uncharacterized lncRNA that was found to be heavily enriched here, and it exhibits high liver specificity relative to other organs (Yu et al., 2014). Moreover, co-expression analysis indicated a significant correlation of LOC102552718 with Itpr1 in a trans pattern (Table 2), and Itpr1 and Itpr2 were significantly downregulated in the MCT treatment group according to the microarray and RT-PCR results (Itpr1: fold change = −3.52875, *p*-value = 0.001393; Itpr2: fold change = −11.0638, *p*-value = 0.008441).

**FIGURE 8:**
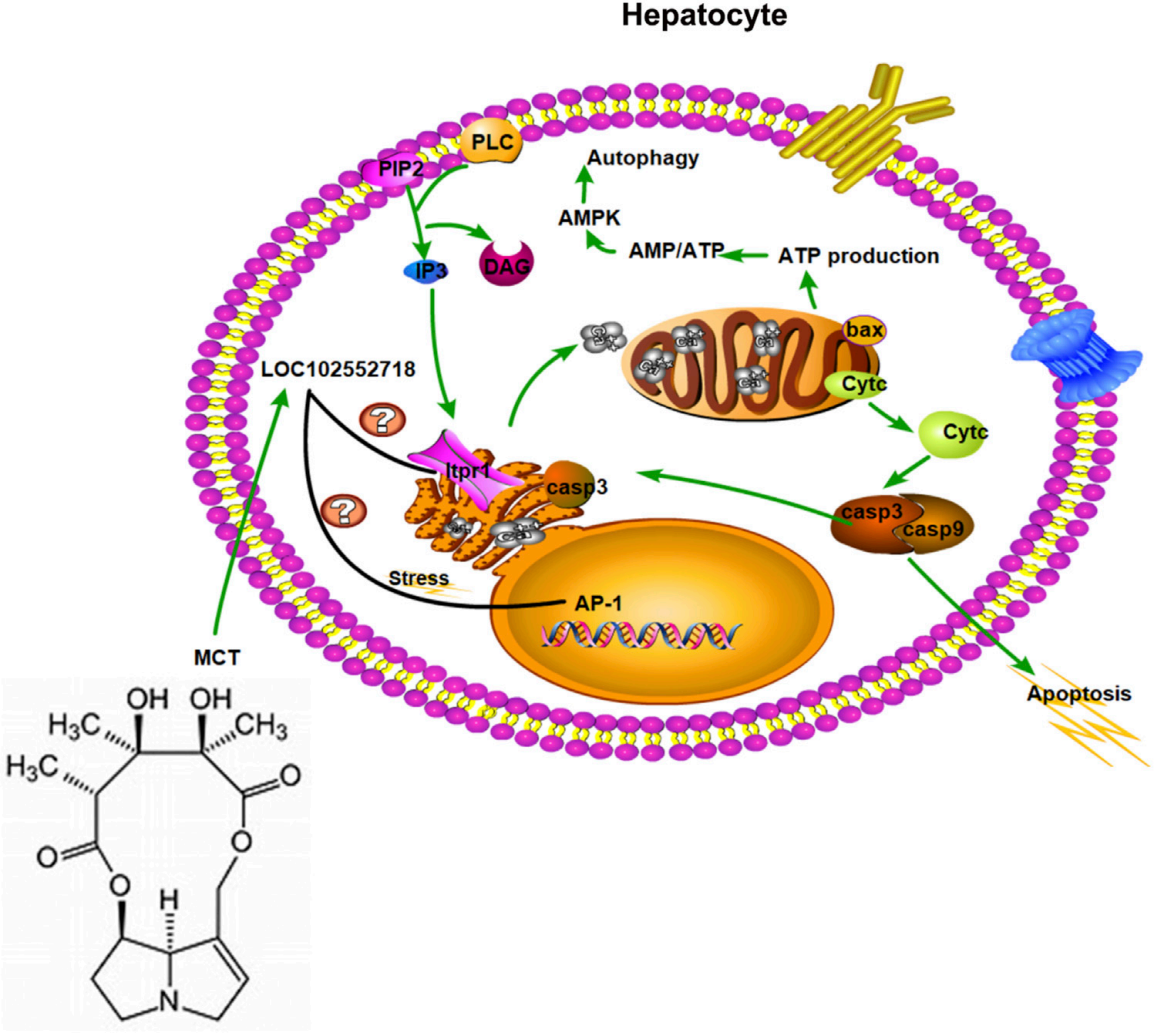
Hypothesized role of *LOC102552718* with Itpr1 in hepatotoxicity induced by monocrotaline.

Inositol 1,4,5-trisphosphate receptors are intracellular calcium channels that are activated by IP3 to release Ca^2+^ from intracellular stores, as well as modulating Ca^2+^ levels (Ivanova et al., 2014). In hepatocytes, the principal intracellular calcium release channel is the Ca^2+^ signaling that results from the activation of Itpr1 and Itpr2 (Khamphaya et al., 2018). Itpr1 plays an essential role in cell proliferation and liver regeneration (Rodrigues et al., 2007); it also regulates liver cell apoptosis (Hanson et al., 2004; Guerra et al., 2011). Various feedback mechanisms have been proposed, which allow Itpr to play a role in amplifying or inhibiting the calcium-dependent apoptotic pathways (Joseph and Hajnóczky, 2007; Rezuchova et al., 2019). For instance, during apoptosis, caspase-3 binds with Itpr1 and cleaves it, leading to suppression of IP3-induced Ca^2+^ release and potentially interfering with the IP3/Ca^2+^ signaling pathway and intracellular Ca^2+^ homeostasis within cells undergoing apoptosis. Moreover, Itpr1 knockout impairs mitochondrial calcium signaling and reduces hepatic triglycerides and lipid droplet formation (Feriod et al., 2017); inhibiting Itpr1 will abrupt these Ca^2+^ signals, thereby weakening mitochondrial functions and lowering ATP production, causing an increase in AMP/ATP ratio, and consequently activating autophagy *via* AMP-activated protein kinase (AMPK) (Kania et al., 2017). Based on the above results, LOC102552718 might play a role in controlling Itpr1 expression in hepatotoxicity induced by MCT (Figure 8). In particular, the microarray results indicated that inositol-tetrakisphosphate 1-kinase (Itpk1) was downregulated, with 3-fold change (*p*-value = 0.0067), upon exposure to MCT at 160 mg/kg for 12 h. In addition, it was co-expressed with LOC102555010 in a trans pattern and with LOC102548189 in a cis pattern.

**FIGURE 9:**
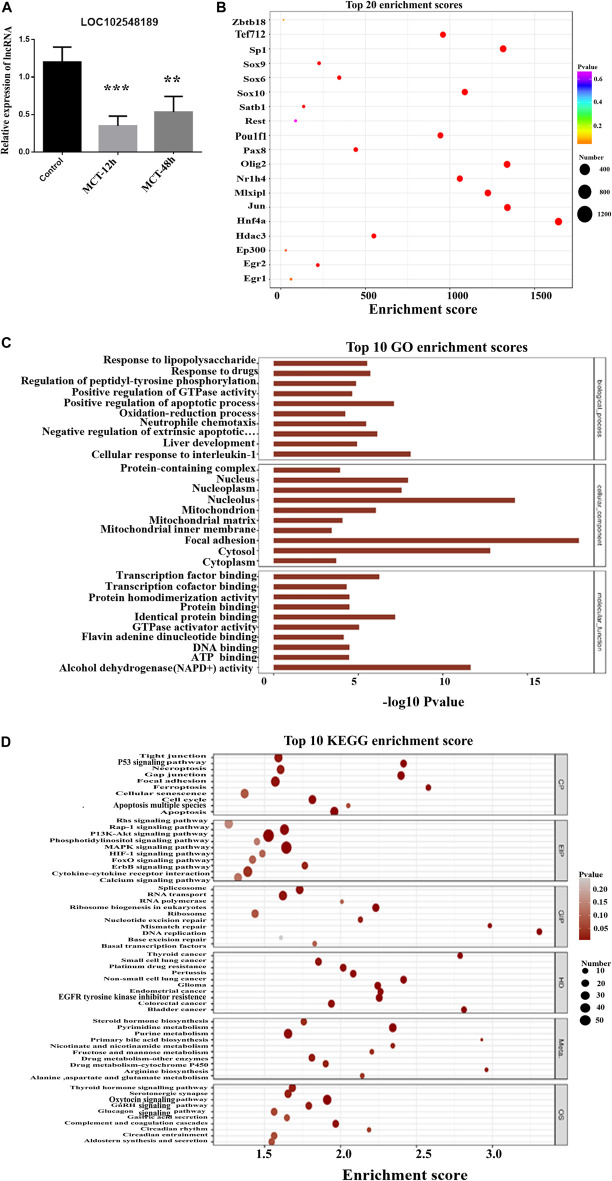
Analysis of LOC102548189 functions in MCT-treated rats. **(A)** Relative expression of LOC102548189 in control and MCT-treated liver tissues (after 12 h and 48 h). Data are presented in the form mean ± SD. *n* = 6 rats per time point. **(B)** Bubble chart of the top 20 enriched transcription factors associated with LOC102548189. **(C)** Top 10 GO enrichment scores for LOC102548189. **(D)** Top 10 KEGG enrichment results for differentially expressed genes co-expressed with LOC102548189. As the *p*-value corresponding to enrichment decreases, the significance level of the enrichment increases. *p ≤ 0.05; **p ≤ 0.01; ***p ≤ 0.001 in comparisons of the MCT-treated group against the control group. Colors differentiate BPs, CCs, and MFs. BP, biological process; CC, cellular component; MF, molecular function.

LOC102548189 (XR_001838555.1) was downregulated 6-fold (*p*-value = 0.0057). It was correlated with Itpk1 in a cis manner, with a correlation coefficient of 0.98. As shown in Figure 9A, RT-PCR for LOC102548189 confirmed the microarray results, in which its level decreased from 12 h MCT treatment onward and remained significantly reduced even at 48 h MCT (HSOS). Based on the functional analysis, one of the topmost enriched pathways corresponding to the mRNAs correlated with LOC102548189 was the phosphatidylinositol 3-kinas/protein kinase (PI3/AKT) pathway. There is evidence that the PI3K/AKT pathway plays a role in the regulation of cell proliferation, the cell cycle, and apoptosis (Rodon et al., 2013). Activation of the PI3K/AKT signaling pathway may regulate antioxidant stress, apoptosis, autophagy, and inflammation *via* downstream related targeted pathways and proteins (Wang et al., 2021). The PI3K/AKT signaling pathway is also activated as a compensatory response to changes in ROS level during liver injury (Ke et al., 2013; Li et al., 2018). In conclusion, LOC102548189 may represent a target for regulation of the PI3K/AKT pathway, thereby helping in the treatment of liver injury induced by MCT; however, further experiments should be carried out to confirm this.

This study provides a foundation for future experiments; the implementation of such future experiments is strongly recommended in order to prove the mechanism proposed here and to determine whether there is translatability from animals to humans, where the lncRNA highlighted here might function as a biomarker for early diagnosis of HSOS caused by monocrotaline.

## 5 Conclusion

In this study, a molecular investigation using microarray and RT-PCR analysis revealed that many essential genes and lncRNAs are altered during MCT-induced hepatotoxicity, especially in early liver injury and HSOS. Although LOC102552718 showed promise as potentially having a regulatory function with Itpr1 in hepatotoxicity, further experiments are recommended to determine the exact mechanisms and biological action of LOC102552718 in this model.

## Data Availability

The datasets presented in this study can be found in online repositories. The names of the repository/repositories and accession number(s) can be found below: https://www. ncbi.nlm.nih.gov/geo/query/acc.cgi?acc=GSE213031, GSE213031.

## References

[B1] Alkan F. Wenzel A. Palasca O. Kerpedjiev P. Rudebeck A. F. Stadler P. F. (2017). RIsearch2: suffix array-based large-scale prediction of RNA-RNA interactions and siRNA off-targets. Nucleic acids Res. 45 (8), e60. 10.1093/nar/gkw1325 28108657 PMC5416843

[B2] Amin K. A. Hassan M. S. Awad el S. T. Hashem K. S. (2011). The protective effects of cerium oxide nanoparticles against hepatic oxidative damage induced by monocrotaline. Int. J. Nanomed. 6, 143–149. 10.2147/IJN.S15308 PMC3026579 21289991

[B3] Ashburner M. Ball C. A. Blake J. A. Botstein D. Butler H. Cherry J. M. (2000). Gene ontology: tool for the unification of biology. The gene ontology Consortium. Nat. Genet. 25 (1), 25–29. 10.1038/75556 10802651 PMC3037419

[B4] Boehm E. Zaganelli S. Maundrell K. Jourdain A. A. Thore S. Martinou J. C. (2017). FASTKD1 and FASTKD4 have opposite effects on expression of specific mitochondrial RNAs, depending upon their endonuclease-like RAP domain. Nucleic acids Res. 45 (10), 6135–6146. 10.1093/nar/gkx164 28335001 PMC5449608

[B5] Caldwell-Kenkel J. C. Thurman R. G. Lemasters J. J. (1988). Selective loss of nonparenchymal cell viability after cold ischemic storage of rat livers. Transplantation 45 (4), 834–836. 10.1097/00007890-198804000-00041 3282369

[B6] Chen D. L. Shen D. Y. Han C. K. Tian Y. (2019). LncRNA MEG3 aggravates palmitate-induced insulin resistance by regulating miR-185-5p/Egr2 axis in hepatic cells. Eur. Rev. Med. Pharmacol. Sci. 23 (12), 5456–5467. 10.26355/eurrev_201906_18215 31298399

[B7] Coppell J. A. Richardson P. G. Soiffer R. Martin P. L. Kernan N. A. Chen A. (2010). Hepatic veno-occlusive disease following stem cell transplantation: incidence, clinical course, and outcome. J. Am. Soc. Blood Marrow Transplant. 16 (2), 157–168. 10.1016/j.bbmt.2009.08.024 PMC3018714 19766729

[B8] Cox T. R. Erler J. T. (2011). Remodeling and homeostasis of the extracellular matrix: implications for fibrotic diseases and cancer. Dis. models Mech. 4 (2), 165–178. 10.1242/dmm.004077 PMC3046088 21324931

[B9] Cursio R. Mari B. Louis K. Rostagno P. Saint-Paul M. C. Giudicelli J. (2002). Rat liver injury after normothermic ischemia is prevented by a phosphinic matrix metalloproteinase inhibitor. FASEB J. Off. Publ. Fed. Am. Soc. Exp. Biol. 16 (1), 93–95. 10.1096/fj.01-0279fje 11709491

[B10] DeLeve L. D. McCuskey R. S. Wang X. Hu L. McCuskey M. K. Epstein R. B. (1999). Characterization of a reproducible rat model of hepatic veno-occlusive disease. Hepatol. (Baltim. Md) 29 (6), 1779–1791. 10.1002/hep.510290615 10347121

[B11] DeLeve L. D. Wang X. Kanel G. C. Ito Y. Bethea N. W. McCuskey M. K. (2003). Decreased hepatic nitric oxide production contributes to the development of rat sinusoidal obstruction syndrome. Hepatol. (Baltim. Md) 38 (4), 900–908. 10.1053/jhep.2003.50383 14512877

[B12] DiStefano J. K. Gerhard G. S. (2022). Long noncoding RNAs and human liver disease. Annu. Rev. Pathol. Mech. Dis. 17 (1), 1–21. 10.1146/annurev-pathol-042320-115255 PMC9183788 34416820

[B13] Djebali S. Davis C. A. Merkel A. Dobin A. Lassmann T. Mortazavi A. (2012). Landscape of transcription in human cells. Nature 489 (7414), 101–108. 10.1038/nature11233 22955620 PMC3684276

[B14] Endo H. Niioka M. Sugioka Y. Itoh J. Kameyama K. Okazaki I. (2011). Matrix metalloproteinase-13 promotes recovery from experimental liver cirrhosis in rats. Pathobiol. J. Immunopathol. Mol. Cell. Biol. 78 (5), 239–252. 10.1159/000328841 21849805

[B15] Feriod C. N. Oliveira A. G. Guerra M. T. Nguyen L. Richards K. M. Jurczak M. J. (2017). Hepatic inositol 1,4,5 trisphosphate receptor type 1 mediates fatty liver. Hepatol. Commun. 1 (1), 23–35. 10.1002/hep4.1012 28966992 PMC5613674

[B16] Friedman S. L. Maher J. J. Bissell D. M. (2000). Mechanisms and therapy of hepatic fibrosis: Report of the AASLD single topic basic research conference. Hepatol. (Baltim. Md) 32 (6), 1403–1408. 10.1053/jhep.2000.20243 11093750

[B17] Geisler S. Coller J. (2013). RNA in unexpected places: Long non-coding RNA functions in diverse cellular contexts. Nat. Rev. Mol. Cell Biol. 14 (11), 699–712. 10.1038/nrm3679 24105322 PMC4852478

[B18] Guerra M. T. Fonseca E. A. Melo F. M. Andrade V. A. Aguiar C. J. Andrade L. M. (2011). Mitochondrial calcium regulates rat liver regeneration through the modulation of apoptosis. Hepatol. (Baltim. Md) 54 (1), 296–306. 10.1002/hep.24367 PMC3125477 21503946

[B19] Hamada T. Fondevila C. Busuttil R. W. Coito A. J. (2008). Metalloproteinase-9 deficiency protects against hepatic ischemia/reperfusion injury. Hepatol. (Baltim. Md) 47 (1), 186–198. 10.1002/hep.21922 17880014

[B20] Hanson C. J. Bootman M. D. Roderick H. L. (2004). Cell signalling: IP3 receptors channel calcium into cell death. Curr. Biol. Cb. 14 (21), R933–R935. 10.1016/j.cub.2004.10.019 15530388

[B21] Hanumegowda U. M. Copple B. L. Shibuya M. Malle E. Ganey P. E. Roth R. A. (2003). Basement membrane and matrix metalloproteinases in monocrotaline-induced liver injury. Toxicol. Sci. Off. J. Soc. Toxicol. 76 (1), 237–246. 10.1093/toxsci/kfg222 12970574

[B22] Harty M. W. Huddleston H. M. Papa E. F. Puthawala T. Tracy A. P. Ramm G. A. (2005). Repair after cholestatic liver injury correlates with neutrophil infiltration and matrix metalloproteinase 8 activity. Surgery 138 (2), 313–320. 10.1016/j.surg.2005.04.009 16153442

[B23] Holoch D. Moazed D. (2015). RNA-mediated epigenetic regulation of gene expression. Nat. Rev. Genet. 16 (2), 71–84. 10.1038/nrg3863 25554358 PMC4376354

[B24] Iimuro Y. Nishio T. Morimoto T. Nitta T. Stefanovic B. Choi S. K. (2003). Delivery of matrix metalloproteinase-1 attenuates established liver fibrosis in the rat. Gastroenterology 124 (2), 445–458. 10.1053/gast.2003.50063 12557150

[B25] Ivanova H. Vervliet T. Missiaen L. Parys J. B. De Smedt H. Bultynck G. (2014). Inositol 1,4,5-trisphosphate receptor-isoform diversity in cell death and survival. Biochim. Biophys. Acta 1843 (10), 2164–2183. 10.1016/j.bbamcr.2014.03.007 24642269

[B26] Jirouskova M. Zbodakova O. Gregor M. Chalupsky K. Sarnova L. Hajduch M. (2012). Hepatoprotective effect of MMP-19 deficiency in a mouse model of chronic liver fibrosis. PloS one 7 (10), e46271. 10.1371/journal.pone.0046271 23056273 PMC3467204

[B27] Joseph S. K. Hajnóczky G. (2007). IP3 receptors in cell survival and apoptosis: Ca2+ release and beyond. Apoptosis Int. J. Program. Cell death 12 (5), 951–968. 10.1007/s10495-007-0719-7 17294082

[B28] Kania E. Roest G. Vervliet T. Parys J. B. Bultynck G. (2017). IP(3) receptor-mediated calcium signaling and its role in autophagy in cancer. Front. Oncol. 7, 140. 10.3389/fonc.2017.00140 28725634 PMC5497685

[B29] Ke B. Shen X. D. Zhang Y. Ji H. Gao F. Yue S. (2013). KEAP1-NRF2 complex in ischemia-induced hepatocellular damage of mouse liver transplants. J. Hepatol. 59 (6), 1200–1207. 10.1016/j.jhep.2013.07.016 23867319 PMC4524560

[B30] Khamphaya T. Chukijrungroat N. Saengsirisuwan V. Mitchell-Richards K. A. Robert M. E. Mennone A. (2018). Nonalcoholic fatty liver disease impairs expression of the type II inositol 1,4,5-trisphosphate receptor. Hepatol. (Baltim. Md) 67 (2), 560–574. 10.1002/hep.29588 PMC5893412 29023819

[B31] Li Y. Tong L. Zhang J. Zhang Y. Zhang F. (2018). Galangin alleviates liver ischemia-reperfusion injury in a rat model by mediating the PI3K/AKT pathway. Cell. Physiol. Biochem. Int. J. Exp. Cell. Physiol. Biochem. Pharmacol. 51 (3), 1354–1363. 10.1159/000495553 30481779

[B32] McKeown C. M. Edwards V. Phillips M. J. Harvey P. R. Petrunka C. N. Strasberg S. M. (1988). Sinusoidal lining cell damage: the critical injury in cold preservation of liver allografts in the rat. Transplantation 46 (2), 178–190. 10.1097/00007890-198808000-00001 3043774

[B33] Mi H. Huang X. Muruganujan A. Tang H. Mills C. Kang D. (2017). PANTHER version 11: expanded annotation data from gene ontology and reactome pathways, and data analysis tool enhancements. Nucleic acids Res. 45 (D1), D183–D189. 10.1093/nar/gkw1138 27899595 PMC5210595

[B34] Neuman M. G. Cohen L. Opris M. Nanau R. M. Hyunjin J. (2015). Hepatotoxicity of pyrrolizidine alkaloids. J. Pharm. Pharm. Sci. Can. des Sci. Pharm. 18 (4), 825–843. 10.18433/j3bg7j 26626258

[B35] Oliva-Vilarnau N. Hankeova S. Vorrink S. U. Mkrtchian S. Andersson E. R. Lauschke V. M. (2018). Calcium signaling in liver injury and regeneration. Front. Med. 5, 192. 10.3389/fmed.2018.00192 PMC6039545 30023358

[B36] Oliveira A. G. Andrade V. A. Guimarães E. S. Florentino R. M. Sousa P. A. Marques P. E. (2015). Calcium signalling from the type I inositol 1,4,5-trisphosphate receptor is required at early phase of liver regeneration. Liver Int. 35 (4), 1162–1171. 10.1111/liv.12587 24814243

[B37] Quinn J. J. Chang H. Y. (2016). Unique features of long non-coding RNA biogenesis and function. Nat. Rev. Genet. 17 (1), 47–62. 10.1038/nrg.2015.10 26666209

[B38] Rezuchova I. Hudecova S. Soltysova A. Matuskova M. Durinikova E. Chovancova B. (2019). Type 3 inositol 1,4,5-trisphosphate receptor has antiapoptotic and proliferative role in cancer cells. Cell Death Dis. 10 (3), 186. 10.1038/s41419-019-1433-4 30796197 PMC6385365

[B39] Rodon J. Dienstmann R. Serra V. Tabernero J. (2013). Development of PI3K inhibitors: Lessons learned from early clinical trials. Nat. Rev. Clin. Oncol. 10 (3), 143–153. 10.1038/nrclinonc.2013.10 23400000

[B40] Rodrigues M. A. Gomes D. A. Leite M. F. Grant W. Zhang L. Lam W. (2007). Nucleoplasmic calcium is required for cell proliferation. J. Biol. Chem. 282 (23), 17061–17068. 10.1074/jbc.M700490200 17420246 PMC2825877

[B41] Roeder E. (1995). Medicinal plants in Europe containing pyrrolizidine alkaloids. Die Pharm. 50 (2), 83–98. 7700976

[B42] Takahashi K. Yan I. Haga H. Patel T. (2014). Long noncoding RNA in liver diseases. Hepatol. (Baltim. Md) 60 (2), 744–753. 10.1002/hep.27043 PMC4110118 24493213

[B43] The Gene Ontology Consortium (2019). The gene ontology resource: 20 years and still GOing strong. Nucleic acids Res. 47 (D1), D330–D338. 10.1093/nar/gky1055 30395331 PMC6323945

[B44] Wang M. Zhang J. Gong N. (2021). Role of the PI3K/akt signaling pathway in liver ischemia reperfusion injury: a narrative review. Ann. Palliat. Med. 11 (2), 806–817. 10.21037/apm-21-3286 35016518

[B45] Wucher V. Legeai F. Hédan B. Rizk G. Lagoutte L. Leeb T. (2017). FEELnc: a tool for long non-coding RNA annotation and its application to the dog transcriptome. Nucleic acids Res. 45 (8), e57. 10.1093/nar/gkw1306 28053114 PMC5416892

[B46] Yang M. Ruan J. Fu P. P. Lin G. (2016). Cytotoxicity of pyrrolizidine alkaloid in human hepatic parenchymal and sinusoidal endothelial cells: Firm evidence for the reactive metabolites mediated pyrrolizidine alkaloid-induced hepatotoxicity. Chemico-Biol. Interact. 243, 119–126. 10.1016/j.cbi.2015.09.011 26365561

[B47] Yevshin I. Sharipov R. Valeev T. Kel A. Kolpakov F. (2017). Gtrd: a database of transcription factor binding sites identified by ChIP-seq experiments. Nucleic acids Res. 45 (D1), D61–D67. 10.1093/nar/gkw951 27924024 PMC5210645

[B48] Yu X. Z. Ji T. Bai X. L. Liang L. Wang L. Y. Chen W. (2013). Expression of MMP-9 in hepatic sinusoidal obstruction syndrome induced by Gynura segetum. J. Zhejiang Univ. Sci. B 14 (1), 68–75. 10.1631/jzus.B1200112 23303633 PMC3542960

[B49] Yu Y. Fuscoe J. C. Zhao C. Guo C. Jia M. Qing T. (2014). A rat RNA-Seq transcriptomic BodyMap across 11 organs and 4 developmental stages. Nat. Commun. 5, 3230. 10.1038/ncomms4230 24510058 PMC3926002

[B50] Zhuang W. Lian G. Huang B. Du A. Xiao G. Gong J. (2018). Pulmonary arterial hypertension induced by a novel method: Twice-intraperitoneal injection of monocrotaline. Exp. Biol. Med. (Maywood, NJ) 243 (12), 995–1003. 10.1177/1535370218794128 PMC6180403 30099957

