# Supplementary material for: Expression profiles of lncRNAs and their possible regulatory role in monocrotaline-induced HSOS in rats
Source: Front Genet. 2023 Jan 26;14:1041266. doi: 10.3389/fgene.2023.1041266 (PMC9909345; doi:10.3389/fgene.2023.1041266)
Supplement: Supplementary file 1 [file Table1.docx]

Expression profiles of lncRNAs and their possible regulatory role in monocrotaline-induced HSOS in rats

**Mohammed Ismail^1,3,^** ^†^ **, Xi Zhang^1,^** ^†^ **, Reham Taha^1^ , Muhanad Elhafiz^1^ , Qianwen Zhang^1^, Bashir A. Yousef^4^ , Xin Huang^1^ , Zhenzhou Jiang^1^ , Luyong Zhang^1,2,^*, Lixin Sun^1,^***

**^1^** Key Laboratory of Drug Quality Control and Pharmacovigilance, China Pharmaceutical University, Nanjing 210009, China.

**^2^**  Centre for Drug Research and Development, Guangdong Pharmaceutical University, Guangzhou 510006, China.

**^3^** Department of Pharmacology, Faculty of Medicine and health science, Dongola University, Dongola, Sudan.

**^4^** Department of Pharmacology, Faculty of Pharmacy, University of Khartoum, Khartoum, Sudan.

^*^**Corresponding Authors**:

Lixin Sun, Key Laboratory of Drug Quality Control and Pharmacovigilance, China Pharmaceutical University, Nanjing 210009, China.

Email: [slxcpu@126.com](mailto:slxcpu@126.com).

Fax: +86 25831142,

Tel: +86 2583271043

Luyong Zhang, Centre for Drug Research and Development, Guangdong Pharmaceutical University, Guangzhou, China.

E-mail: [lyzhang@cpu.edu.cn](mailto:lyzhang@cpu.edu.cn)

Fax: + (86) 2039352086,

Tel: + (86) 2039352100

**^†^** These authors contributed equally to this work and share first authorship

| Gene Name | Primer Sequence |  |
| --- | --- | --- |
| CD32 | F GCCGGTCTCCTCCATTCGGC  R GCCTGTGCCTGCAGCTGACT |  |
| Lyve1 | F GGTCTGTAAGGTTCTGGGACTG  R AGACGTTGTGGAGTCTGAGGAT |  |
| Stab2 | F TGCTTCAAATGGGCTTGTGC  R GCCACGTTGTTTGCCAGAAT |  |
| Gapdh | F GCATCTTCTTGTGCAGTGCC  R TACGGCCAAATCCGTTCACA |  |
| LOC0960120 | F GGCCGGGAGTATTAGATACTGA  R CTGCATGAACGTATACACGC |  |
| LOC108350110 | F GGGAGTAACTAGGCATCCG  R CCAGAAAGGGAAGTACGCA |  |
| LOC102553561 | F CGTCTCCACAATGATGAGC  R CCTATTGTTCCTATCACAGCG |  |
| LOC102552718 | F ACAGCAAAGACACGGATT  R GGCACCACTACTTGTTTAGG |  |
| Wrd74 | F TTGGAACTGAAGGTGCGGAA  R TTCTGTGACCCAGGGAGGAA |  |
| Slc3a2 | F GGTACCTGCAGAAACGGTGA  R GCTAACTAGCCTCACGGGAC |  |
| Itpr1 | F GTG GAG GTT TCA TCT GCA AGC  R GCT TTC GTG GAA TAC TCG GTC |  |
| Itpr2 | F GCT CTT GTC CCT GAC ATT G  R CCC ATG TCT CCA TTC TCA TAG C |  |
| LOC103692937 | F TTGCATCTCAACACAGTACATT  R TGGCACACTAAGGGTAGAAG |  |
| LOC102554284 | F AAAGAACAAAGGCCATATCTCC  R GGACCTCAGAGAAGCAATCA |  |

**Supplemental Table S1; Primer sequences**

**Supplemental Table S2:**

| **Group name** | **Type** | **up** | **down** | **Total** |
| --- | --- | --- | --- | --- |
| Comp1_M_vs _C_FC2P 0.05 | mRNA | 1836 | 2385 | 4221 |
| Comp1_M_vs _C_FC2P 0.05 | ncRNA | 113 | 63 | 176 |

The total number of differential expression mRNAs and lncRNAs both upregulated and downregulated for the control and MCT treatment group.

**Supplemental Table S3:**

Correlation between the expressions of the differential LOC102552718 and differential genes expression**;** Use Pearson correlation test (Pearson) to calculate the correlation between the expressions of the differential LOC102552718 and differential gene expression, and select the correlation pair with a correlation coefficient ≥ 0.9 and a p-value of less than or equal to 0.05.

| ***lncRNA symbol*** | ***LncRNA-id*** | ***lncRNA chrom*** | ***Gene symbol*** | ***mRNA-id*** | ***mRNA chrom*** | ***Correlation Coefficient*** | ***p-value*** |
| --- | --- | --- | --- | --- | --- | --- | --- |
| LOC102552718 | XR_357275.3 | Chr9 | Itpr1-X6 | XM_008763172.2 | Chr4 | 0.999302 | 7.31E-07 |
|  |  |  | Itpr1-X10 | XM_008763176.2 | Chr4 | 0.999226 | 8.98E-07 |
|  |  |  | Prex2 | NM_001107899.1 | Chr5 | 0.999095 | 1.23E-06 |
|  |  |  | Itpr1-X3 | XM_008763169.2 | Chr4 | 0.998915 | 1.77E-06 |
|  |  |  | Slc16a10 | XM_008772975.2 | Chr20 | 0.99849 | 3.42E-06 |
|  |  |  | Itpr1- X1 | XM_008763167.2 | Chr4 | 0.998484 | 3.44E-06 |
|  |  |  | Itpr1-1 | NM_001007235.2 | Chr4 | 0.998361 | 4.03E-06 |
|  |  |  | Atxn7l1 | XM_006240014.3 | Chr6 | 0.998352 | 4.07E-06 |
|  |  |  | Peak1 | NM_001108149.1 | Chr8 | 0.998301 | 4.33E-06 |
|  |  |  | Atxn7l1 | XM_008764632.2 | Chr6 | 0.998286 | 4.40E-06 |
|  |  |  | Itpr1-X12 | XM_008763177.2 | Chr4 | 0.998063 | 5.62E-06 |
